# Effect of Weight-Shifting Practice Using Auditory Feedback on Postural Control in Patients With Body Lateropulsion: A Single-Case Experimental Design

**DOI:** 10.7759/cureus.77201

**Published:** 2025-01-09

**Authors:** Daiki Abe, Tatsuya Igarashi, Satoshi Yamamoto, Yohei Tomioka

**Affiliations:** 1 Department of Rehabilitation, IMS Itabashi Rehabilitation Hospital, Tokyo, JPN; 2 Department of Physical Therapy, Faculty of Health Science Technology, Bunkyo Gakuin University, Saitama, JPN

**Keywords:** auditory feedback, body lateropulsion, postural control, proprioception, stabilometer, subjective visual vertical

## Abstract

Body lateropulsion (BL) is a postural control disorder commonly associated with unilateral brainstem or cerebellar lesions. Patients with BL exhibit a tendency to lean toward one side and experience difficulty maintaining stable standing and walking. Although exercises focused on visual or somatosensory cues have been proposed, no standardized interventions have been established. Auditory feedback has emerged as a promising new approach, as it can complement visual and somatosensory inputs to improve balance. This single-case study investigated whether incorporating auditory feedback into standing weight-shifting exercises could enhance postural control in patients with BL.

A man in his 60s with BL following a left cerebellar hemorrhage participated in an ABA (A: control phase, B: intervention phase) single-case study design. Each phase (A1, B, A2) lasted seven days, with weight-shifting exercises performed daily. During the A1 and A2 phases, the patient performed weight-shifting exercises without auditory feedback. In the B phase, auditory feedback was incorporated using a shoe-based load meter. Primary outcomes included center of pressure (COP) measures, COP velocity, perimeter area, and mediolateral COP position, recorded under eyes-open and eyes-closed conditions. Secondary outcomes included the Scale for the Assessment and Rating of Ataxia (SARA), the Berg Balance Scale (BBS), and subjective visual vertical (SVV).

Compared to the A1 phase, the B phase demonstrated significant improvements in COP velocity, perimeter area, and mediolateral COP position. These improvements were maintained after auditory feedback was removed in the A2 phase. Although ataxia and balance ability improved over time, the changes did not exceed the minimal detectable change (MDC) or the minimal clinically important difference (MCID). The SVV deviation showed slight improvement but remained outside the normal range.

Incorporating auditory feedback into weight-shifting exercises improved postural stability in a patient with BL. These findings suggest that auditory cues may facilitate proprioceptive reweighting and motor learning in postural control, independently of improvements in vestibular function or visual vertical perception. Further studies with larger sample sizes are needed to validate these results and elucidate the underlying mechanisms.

## Introduction

Body lateropulsion (BL) is a postural control disorder caused by unilateral lesions in the brainstem or cerebellum [1,2]. It is characterized by the body tilting to one side during standing or walking [1]. Lateropulsion, also referred to as “pusher syndrome,” “pusher behavior,” or “contraversive pushing,” is a clinical symptom arising from damage to areas such as the thalamus or insular cortex [3-5]. It is defined by active pushing of the body across the midline toward the more affected side and/or active resistance to weight shifts toward the less affected side [6]. Although both lateropulsion and BL share the characteristic of leaning toward one side, the pushing behavior and resistance to corrective movements observed in lateropulsion are absent in BL. Therefore, lateropulsion and BL represent distinct pathological conditions, and this study focuses specifically on BL.

BL primarily occurs due to lesions in the lateral medulla [7], with the main causes being damage to the lateral vestibulospinal tract and the dorsal spinocerebellar tract [8-10]. It has also been reported that BL can result from lesions in the cerebellum [2,11]. In cerebellar lesions, damage to the fastigial nucleus or the inferior cerebellar peduncle has been reported to cause dysfunction in the vestibulospinal and reticulospinal tracts, leading to the development of BL [11]. Furthermore, impairments in the dorsal spinocerebellar tract caused by cerebellar damage may exacerbate BL by resulting in unconscious proprioceptive deficits [11]. Patients with BL often exhibit subjective visual vertical (SVV) deviation, which reflects an imbalance in the vestibular sensory system [1,11]. One study reported that patients with BL required approximately 108 days to achieve an upright standing posture without tilting [1]. Regarding gait, it has been reported that patients with BL need one to three months to walk in a straight line without assistance [12]. BL impairs postural control and walking ability, significantly disrupting the daily lives of affected patients.

Regarding the postural control of patients with BL, Matsuo et al. reported that patients with BL exhibit lateral deviation and increased center of pressure (COP) velocity, indicating impaired postural control [13]. A positive correlation between SVV deviation and gait asymmetry in patients with BL has also been observed [14]. Postural control relies on reweighting visual, vestibular, and somatosensory inputs, which are essential for maintaining balance [15,16]. Based on this principle, physical therapy approaches focusing on visual vertical reference frames and the reweighting of somatosensory inputs have been proposed for patients with BL [13,14,17]. However, these approaches have not been sufficiently validated, and no standardized physical therapy protocol for BL has been established.

In contrast, auditory feedback has emerged as a novel approach to improving postural control. Unlike visual and somatosensory inputs, auditory feedback is independent of environmental constraints. Previous research has demonstrated the effectiveness of auditory feedback in enhancing postural control [18-21]. Studies have also shown that auditory feedback improves postural stability in patients with vestibular disorders [22]. Its efficacy is believed to stem from its ability to complement visual and somatosensory inputs, promoting multisensory integration [21,22]. Additionally, auditory feedback has been reported to activate brain regions associated with proprioceptive processing [23,24]. Ghai et al. further reported that auditory feedback significantly enhances knee proprioception, with improvements persisting even 24 hours after training [25,26]. These findings suggest that auditory feedback facilitates accurate proprioceptive perception, supporting motor control and contributing to motor learning [19].

In patients with BL, compensation through vestibular is challenging due to biases in the SVV. Furthermore, deviation in the SVV, which indicates an impairment in the perception of visual verticality, may contribute to difficulties in postural control when visual cues are absent [17]. Consequently, auditory feedback may assist in reweighting proprioceptive inputs and support postural control. However, no studies have specifically investigated the use of auditory feedback interventions for patients with BL.

Building upon these findings, this study hypothesized that standing weight-shifting exercises incorporating auditory feedback would improve postural control more effectively than exercises without auditory feedback in patients with BL. To test this hypothesis, we examined the effects of weight-shifting exercises incorporating auditory feedback on postural control using a single-case experimental design.

## Case presentation

Participant

**Figure 1:**
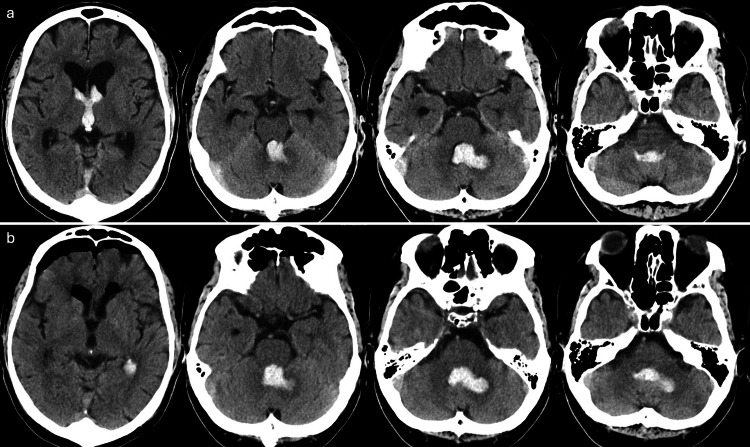
CT imaging of head at onset and postoperatively. a: This is a CT image taken at onset, showing a hematoma in the left cerebellum. The hematoma has ruptured into the ventricular system, with blood also present in the third ventricle. b: This is a CT image obtained after craniotomy for hematoma removal and third ventriculostomy. Although the hematoma in the third ventricle has been removed, the hematoma in the cerebellum remains.

The case involves a man in his 60s who experienced a left cerebellar hemorrhage (Figure 1) and, on the same day, developed hydrocephalus caused by intraventricular hemorrhage. He underwent a craniotomy for hematoma removal and a third ventriculostomy. Prior to the onset of his condition, he was independent in walking and lived with his wife, younger brother, two sons, and two daughters, making a total of seven family members. His medical history included hypertension and hepatitis C. Approximately two years before admission, he began experiencing numbness in his fingers and toes. During this hospitalization, he was newly diagnosed with untreated diabetes mellitus, and pharmacological treatment was initiated. Postoperatively, orthostatic hypotension was observed, which required adjustments to his antihypertensive medication. Gradual mobilization was initiated, and 57 days after the onset of the cerebellar hemorrhage, he was transferred to a rehabilitation hospital for recovery.

At the time of transfer, no significant paralysis, sensory deficits, or cognitive impairments were observed. The patient scored 7 points on the Scale for the Assessment and Rating of Ataxia (SARA), indicating mild ataxia. The trunk coordination test was graded as II (i.e., in a seated position, external stimuli applied by the examiner reveal trunk instability and diminished equilibrium responses), and the Berg Balance Scale (BBS) score was 47. On visual inspection, neither spontaneous nystagmus nor gaze-evoked nystagmus was observed. The patient occasionally complained of floating dizziness but did not report rotational vertigo. He was able to walk independently without a cane under supervision, with a gait speed of 0.74 m/s. During walking, he reported a sensation of being pulled to the left and exhibited postural tilting and instability toward the left. The Burke Lateropulsion Scale score was 0, indicating no resistance to postural tilting, which was consistent with BL. The grading of lateropulsion was classified as Grade I [1]. The SVV was deviated to the right by 4.5 ± 0.53°. The Functional Independence Measure (FIM) score was assessed at 82.

This study was approved by the Ethics Committee of our institution (No. B-51). In accordance with the Declaration of Helsinki, the purpose of the study was explained to the participant both orally and in writing, and informed consent was obtained.

Study design

**Figure 2:**
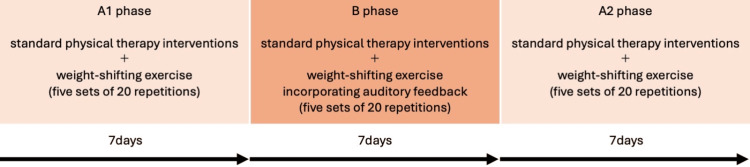
Timeline of the ABA single case design. A: control phase, B: intervention phase

An ABA (A: control phase, B: intervention phase) single-case design study was conducted starting 57 days after the onset of cerebellar hemorrhage, with each phase lasting seven days (Figure 2). The A1, B, and A2 phases each spanned seven days, with one 60-minute session conducted per day, resulting in a total of seven sessions per phase and 21 sessions overall. Throughout the study period, physical therapy interventions were administered individually by three physical therapists with two to six years of clinical experience. For the two physical therapists other than the author, the intervention procedures, measurement items, and methods were thoroughly explained orally in advance, and written documentation was recorded in the patient’s medical records.

In all phases, weight-shifting exercises were performed in a standing position with the feet placed approximately hip-width apart on a linoleum floor. The patient’s arms were positioned at their sides, and they were instructed to look straight ahead. No specific instructions were provided regarding the speed of weight shifting. The exercise involved shifting weight from the right side to the midline, with the timing of the weight shifts indicated by counting repetitions. During the A1 and A2 phases, the weight-shifting exercise was performed in five sets of 20 repetitions each. The remaining time was allocated to standard physical therapy interventions, including balance and gait training. During the B phase, the weight-shifting exercise was similarly conducted in five sets of 20 repetitions, but auditory feedback was incorporated. The remaining time was again dedicated to standard physical therapy interventions, such as balance and gait training. Breaks were provided as needed between sets.

During the A1 and A2 phases, rightward weight-shifting exercises were performed using flat-soled shoes appropriate for the patient’s size. The patient was instructed as follows: “Shift your weight as far to the right as you can without losing balance. Keep your shoulders parallel to the ground while shifting. After shifting, promptly return to the midline.”

**Figure 3:**
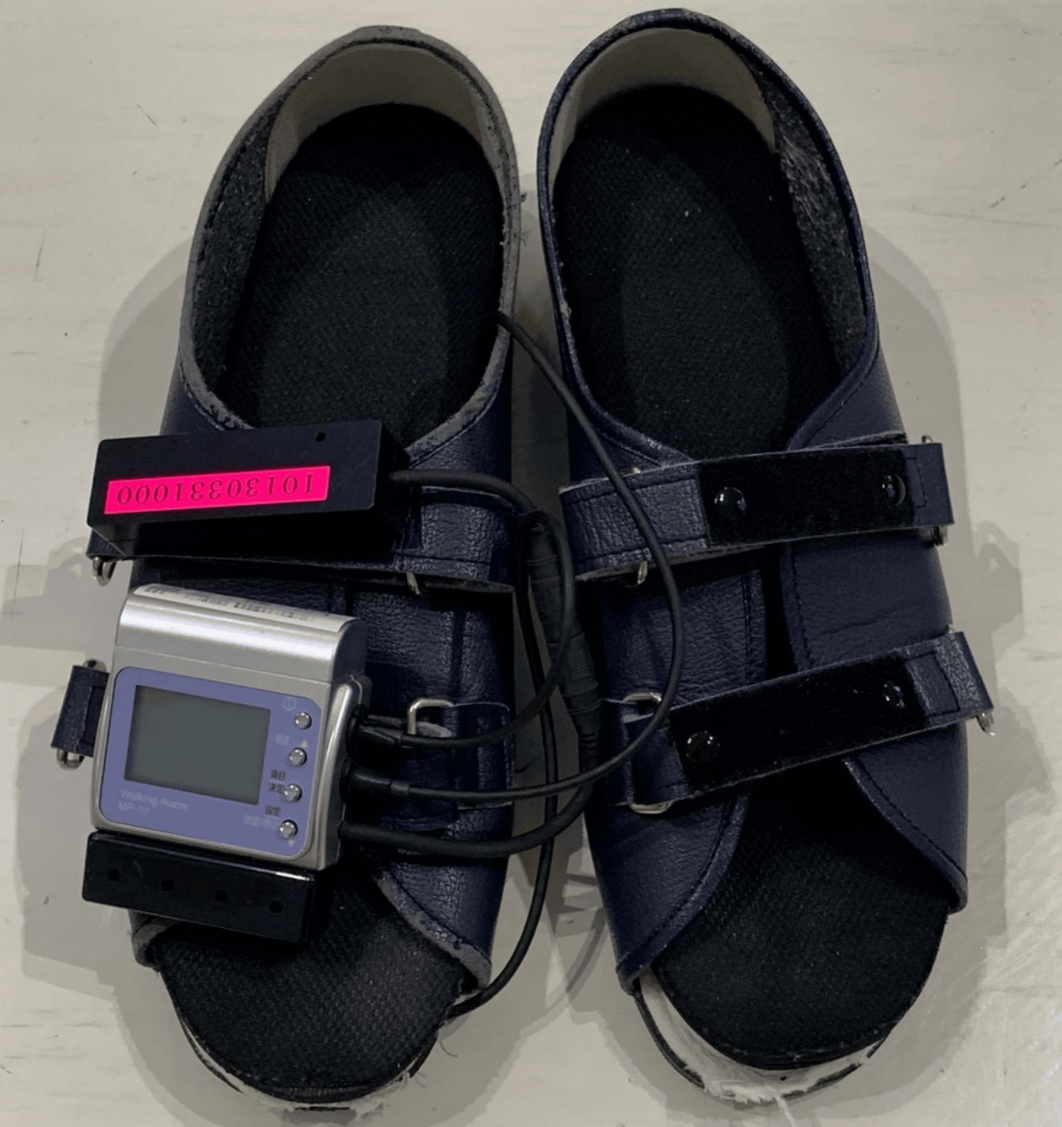
Shoe-based lower-limb load meter. When weight is applied to either side, an alarm sounds from the speaker of the main unit. If upper and lower limits are set, the alarm sounds intermittently when the lower limit is exceeded. As the upper limit is approached, the rhythm of the alarm quickens, and a continuous sound is emitted when the upper limit is exceeded.

In the B phase, in addition to standard physical therapy, standing weight-shifting exercises incorporating auditory feedback were performed. These exercises were conducted using a shoe-based lower-limb load meter (Walking Alarm MP-10; Anima, Tokyo, Japan) (Figure 3). The device was programmed to emit intermittent sounds when the right leg bore more than 80% of the body weight and continuous sounds when the weight exceeded 90%. The sound was emitted from a speaker attached to the shoe at a volume perceivable to the patient. The patient was instructed as follows: “Shift your weight to the right until the sound activates. Keep your shoulders parallel to the ground while shifting. After shifting, promptly return to the midline.”

Evaluation

The primary evaluation parameter was postural control in a standing position, assessed using a stabilometer (Gravicorder G-620; Anima, Tokyo, Japan). Measurements were taken daily after each session during the A1, B, and A2 phases by the therapist conducting that session. The assessment included two conditions: eyes-open and eyes-closed. For each condition, the patient maintained a static standing position on the stabilometer for 30 seconds. The standing posture required the feet to be together and the arms positioned at the sides of the body. Measurements began only after confirming that the patient was completely still. The stabilometer’s sampling frequency was set to 20 Hz, and the following parameters were calculated for each condition: COP velocity, perimeter area, and mediolateral COP position. Positive mediolateral COP values were interpreted as a deviation to the right, while negative values indicated a deviation to the left.

The secondary evaluation parameters included the SARA score, the BBS score, and the SVV. These assessments were conducted four times: at baseline and at the end of the A1, B, and A2 phases. All evaluations were performed by the same physical therapist; however, blinding was not implemented. The SARA was used to assess the severity of cerebellar ataxia, with a maximum score of 40 points, where higher scores indicate greater severity of ataxia [27]. The BBS was used to evaluate the patient’s balance ability, with a maximum score of 56 points, where higher scores reflect better balance ability [28]. The SVV was used to assess the patient’s visual vertical perception and vestibular system imbalance. The bucket method [29] was employed for the SVV assessment. A total of eight trials were conducted, starting from a tilted position of 45° to the left or right (four trials each), and the average value was calculated. We ensured that the patient’s head remained upright during each trial. Positive SVV values were interpreted as deviations to the right of vertical, while negative values indicated deviations to the left.

Statistical analysis

The values of each parameter during each phase were plotted to visually assess the patient’s COP velocity, perimeter area, and mediolateral COP position. As no significant trends were observed during the A or B phases, the effect sizes for COP velocity, perimeter area, and mediolateral COP position under each condition were calculated using Tau-U [30]. Tau-U is a non-overlap measure that accounts for trends during the baseline phase. Effect sizes are categorized as follows: 0.00-0.20 indicates a small change, 0.20-0.60 indicates a moderate change, 0.60-0.80 indicates a large change, and 0.80-1.00 indicates a very large change [31]. The Tau-U scores were calculated using the Tau-U Calculator (Single Case Research™), with the significance level set at 5%.

Results

The patient completed the intervention without any adverse events. The progression of the SARA, BBS, and SVV scores is shown in Table 1-3. Changes in the SARA and BBS scores were observed throughout the study period. The SVV showed a decreasing trend throughout the study.

**Table 1:** Progress of Scale for the Assessment and Rating of Ataxia (SARA). SARA was used to assess the severity of cerebellar ataxia, with a maximum score of 40 points, where higher scores indicate greater severity of ataxia [27]. In patients with ataxic stroke, a score of 8 or below is considered the cutoff value for independent walking [32].

	Baseline	A1	B	A2
Gait	3	2	2	1
Stance	2	1	0	0
Sitting	0	0	0	0
Speech disturbance	0	0	0	0
Finger chase	0.5	0.5	0	0
Nose-finger test	0.5	0	0	0
Fast alternating hand movements	0.5	0.5	0	0
Heel-shin slide	0.5	0	0	0
Total	7	4	2	1

**Table 2:** Progress of Berg Balance Scale (BBS). BBS was used to evaluate the patient’s balance ability, with a maximum score of 56 points, where higher scores reflect better balance ability [28]. In patients with stroke, cutoff scores for fall risk have been reported to range from 46.5 to 50.5 [33].

	Baseline	A1	B	A2
Sitting to standing	4	4	4	4
Standing unsupported	4	4	4	4
Sitting unsupported	4	4	4	4
Standing to sitting	4	4	4	4
Transfers	4	4	4	4
Standing with eyes-closed	3	4	4	4
Standing with feet together	4	4	4	4
Reaching forward with outstretched arm	4	4	4	4
Retrieving object from floor	3	4	4	4
Turning to look behind	4	4	4	4
Turning 360 degrees	3	4	4	4
Placing alternate foot on step	2	2	3	4
Standing with one foot in front	3	3	3	3
One leg stand	1	1	2	3
Total	47	50	52	54

**Table 3:** Progress of subjective visual vertical. SVV: subjective visual vertical. SVV was used to assess the patient’s visual vertical perception and vestibular system imbalance. SVV deviation remained outside the normal range of 2.5° [34].

	Baseline	A1	B	A2
SVV, °	4.5±0.5	3.75±1.28	3.38±1.77	3.25±1.49

**Figure 4:**
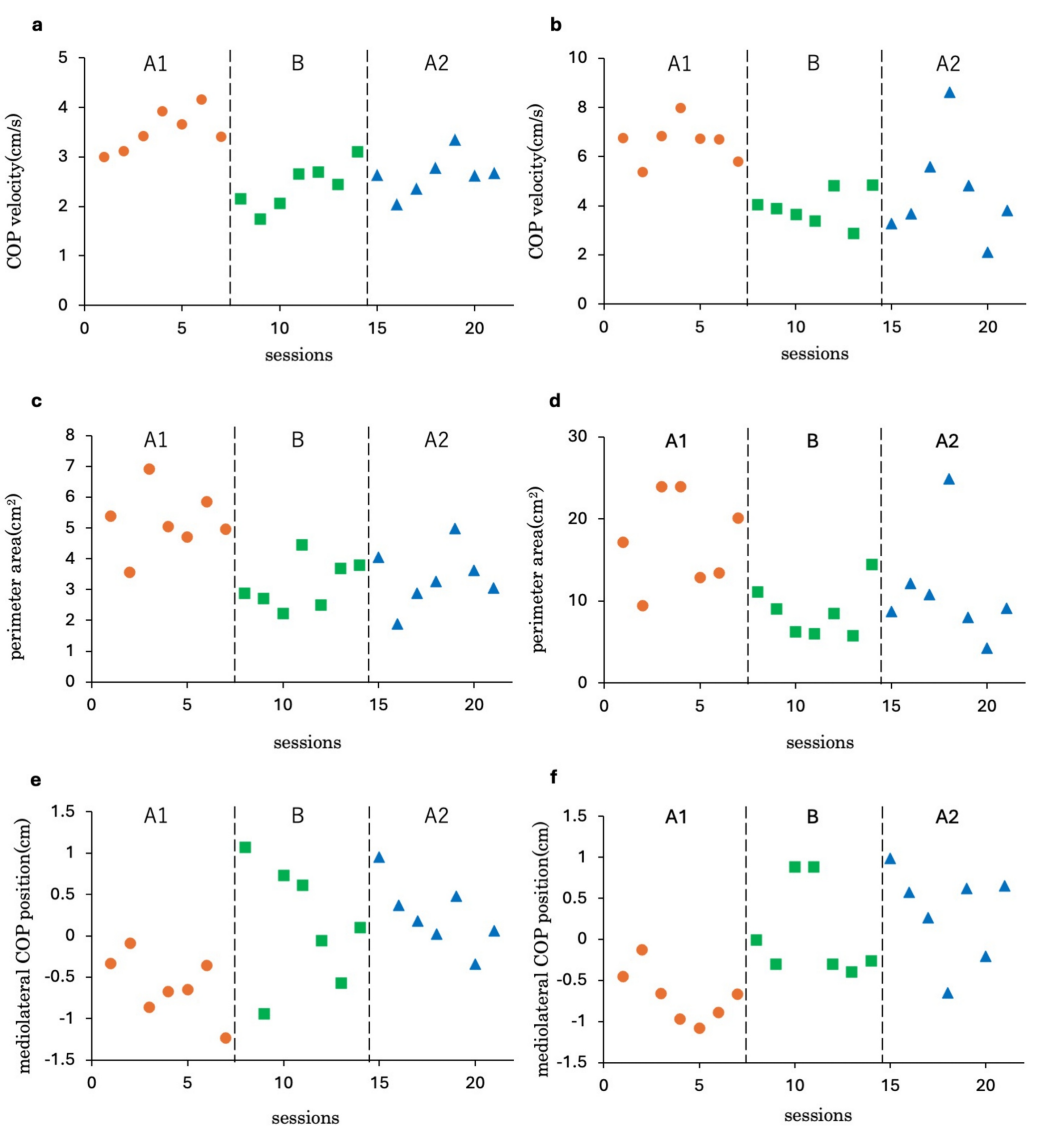
COP velocity, peripheral area and mediolateral COP position for each phase. a: COP velocity (eyes-open); b: COP velocity (eyes-closed); c: peripheral area (eyes-open); d: peripheral area (eyes-closed); e: mediolateral COP position (eyes-open); f: mediolateral COP position (eyes-closed) COP: center of pressure; A1: first control phase; B: intervention phase; A2: second control phase

The time-series changes and Tau-U results for COP velocity, perimeter area, and mediolateral COP position under each condition are presented in Figure 4 and Table 4. Figure 4a illustrates the COP velocity under the eyes-open condition. Significant differences were observed between the A1 and B phases, as well as between the A1 and A2 phases, with very large effect sizes in both cases. Under the eyes-closed condition, significant changes were also observed between the A1 and B phases, as well as between the A1 and A2 phases, with very large and large effect sizes, respectively (Figure 4b). However, no significant differences were observed between the B and A2 phases for COP velocity under either the eyes-open or eyes-closed conditions.

**Table 4:** Results of Tau-U analysis for COP velocity, perimeter area, and mediolateral COP position. COP: center of pressure; A1: first control phase; B: intervention phase; A2: second control phase P-value was calculated using Tau-U.

		A1-B		B-A2		A1-A2	
		Tau-U	P-value	Tau-U	P-value	Tau-U	P-value
COP velocity	eye open	-0.96	0.003	0.22	0.482	-0.92	0.004
	eye closed	-1.00	0.002	0.10	0.749	-0.67	0.035
Perimeter area	eye open	-0.88	0.006	0.16	0.609	-0.79	0.001
	eye closed	-0.84	0.008	0.22	0.482	-0.63	0.048
Mediolateral COP position	eye open	0.63	0.048	0.06	0.848	0.92	0.004
	eye closed	0.84	0.009	0.26	0.406	0.88	0.006

Figures 4c, 4d show the results for the perimeter area. Under the eyes-open condition, a significant decrease was observed in the B and A2 phases compared to the A1 phase. The effect size for the B phase relative to the A1 phase was very large, while the effect size for the A2 phase was large (Figure 4c). Similarly, under the eyes-closed condition, significant differences were observed in the B and A2 phases compared to the A1 phase. The effect sizes for the B and A2 phases relative to the A1 phase were categorized as very large and large, respectively (Figure 4d). However, no significant changes were observed between the B and A2 phases under either the eyes-open or eyes-closed condition.

Figures 4e, 4f show the results for the mediolateral COP position. Under the eyes-open condition, significant changes were observed in the B and A2 phases compared to the A1 phase. The effect size for the B phase relative to the A1 phase was large, while the effect size for the A2 phase was very large (Figure 4e). Similarly, under the eyes-closed condition, significant changes were observed between the A1 and B phases and between the A1 and A2 phases. The effect sizes for the B and A2 phases relative to the A1 phase were both categorized as large (Figure 4f). However, no significant differences were observed between the B and A2 phases under either the eyes-open or eyes-closed condition.

## Discussion

This study employed a single-case design to investigate the effects of standing weight-shifting exercises incorporating auditory feedback on postural control in patients with BL, compared to exercises without auditory feedback. The results demonstrated that during the B phase, where auditory feedback was introduced, significant improvements were observed in COP velocity, perimeter area, and mediolateral COP position compared to the A1 phase, which involved exercises without auditory feedback. These findings suggest that standing weight-shifting exercises with auditory feedback may be an effective intervention for improving postural control in patients with BL. In the A2 phase, significant improvement and a large effect size were also observed compared to the A1 phase; however, no significant difference was found between the B and A2 phases. This suggests two possibilities: first, that the effects achieved during the B phase were maintained through the A2 phase; and second, that the changes observed in the A2 phase may have been influenced by natural recovery over time.

Our findings are generally consistent with previous studies that have demonstrated the effectiveness of auditory feedback in improving postural stability in both healthy adults and patients with vestibular dysfunction [18-22]. Dozza et al. [21] reported that auditory feedback reduces sway, particularly under conditions such as eyes-closed or standing on a rubber foam surface in healthy adults. Similarly, in patients with vestibular dysfunction, auditory feedback has been shown to enhance postural stability [22]. Based on the results of this study, it is suggested that auditory feedback may also contribute to improving postural control in patients with BL.

Although the SARA and BBS scores showed the most significant improvements from baseline to the A1 phase, they continued to improve throughout the study period. Given that motor function in patients with strokes is known to improve significantly within the first three months after onset [35], the improvements in ataxia and balance ability observed throughout the study period in this case may largely reflect the natural course of recovery. However, in each phase, the changes did not exceed the minimal detectable change (MDC) of 3.5 points for SARA in spinocerebellar degeneration [36], the MDC of 6.9 points for BBS in patients with stroke [37], or the minimal clinically important difference (MCID) of 4 points for BBS [38]. This indicates that the observed improvements in ataxia and balance ability during each phase were neither true improvements nor clinically meaningful. That said, significant improvements in postural control were observed in the B and A2 phases compared to the A1 phase. These results suggest that the observed improvements in postural control were not solely attributable to the natural course of recovery in ataxia or balance ability but were potentially influenced by the weight-shifting exercises incorporating auditory feedback.

In this case, damage to the inferior cerebellar peduncle was suspected (Figure 1), and BL was considered to have occurred on the lesion side due to dysfunction of the vestibulospinal and reticulospinal tracts, as well as impairments in unconscious proprioception [11]. However, SVV tilt was observed on the side opposite the lesion, resulting in a discrepancy between the directions of SVV deviation and BL [11,39]. This discrepancy is thought to result from the release of inhibition in the ipsilateral vestibular nucleus caused by the lesion in the inferior cerebellar peduncle, leading to increased resting activity of the ipsilateral vestibular nerve and tilting of the SVV toward the side opposite the lesion [39]. In patients with BL, the SVV often improves over time [1]. In this case, the SVV showed a decreasing trend throughout the study; however, the changes remained within the margin of error. Additionally, the SVV deviation remained outside the normal range of 2.5° [34]. This finding suggests that weight-shifting exercises incorporating auditory feedback may not effectively reduce SVV deviation. Nevertheless, as SVV deviation reflects vestibular system imbalance [1,11], the improvements in postural control observed during the B and A2 phases were likely not attributable to vestibular system recovery or vestibular compensation. Instead, these improvements may have been driven by the weight-shifting exercises incorporating auditory feedback.

In this study, postural control improved not only under the eyes-open condition but also under the eyes-closed condition during the B phase, and these improvements were maintained through the A2 phase. This suggests that the patient developed a postural control strategy that does not rely on vision. Similarly, previous studies [21,22] have reported reductions in sway under eyes-closed conditions facilitated by auditory feedback. These findings indicate that standing weight-shifting exercises incorporating auditory feedback may promote the learning of postural control strategies that are less dependent on vision.

Furthermore, auditory feedback has been shown to promote the activation of extensive networks related to proprioceptive information [23,24] and significantly enhance the perception of knee proprioception [25,26]. Therefore, auditory feedback is suggested to contribute to improvements in standing postural control by facilitating the learning of postural control strategies that utilize proprioceptive information [19]. Based on these findings, the improvements in postural control observed in this study were likely not attributable to visual or vestibular inputs but rather to the learning of postural control strategies involving proprioceptive information facilitated by auditory feedback. In this case, it is considered that damage to the dorsal spinocerebellar tract caused by a lesion in the inferior cerebellar peduncle may have led to impairments in unconscious proprioception (Figure 1). Therefore, auditory feedback might have compensated for this deficit in unconscious proprioception. Additionally, Ghai et al. reported that the enhancement of knee proprioception through auditory feedback is maintained even 24 hours after training [27,28]. Consequently, the sustained effects observed during the A2 phase in this study suggest that the learning effects induced by auditory feedback were retained.

 Matsuo et al. reported that COP velocity is increased in patients with BL [13]. Increased COP sway velocity has been shown to reflect greater reliance on voluntary postural control [40]. In this case, COP velocity significantly decreased after performing weight-shifting exercises incorporating auditory feedback. In a previous study by Nakamura et al. [17], interventions emphasizing proprioceptive input were found to reduce sway velocity, which was hypothesized to result from a reduction in excessive voluntary control. Similarly, in this study, the reduction in sway velocity may have been caused by a decrease in excessive voluntary control.

The limitations of this study include the fact that it involved only a single case and that the intervention period was relatively short. The present case had cerebellar lesions and exhibited a dissociation between postural tilt and SVV deviation [11,39]. In contrast, it has been reported that in cases of BL caused by lateral medullary infarction, postural tilt and SVV deviation are consistent [14]. This suggests that the mechanisms underlying the development of BL may differ between patients with medullary lesions and those with cerebellar lesions [2]. Therefore, the results of this study may not necessarily apply to patients with BL caused by medullary lesions. Additionally, the extent to which postural control relied on proprioceptive reweighting or whether motor learning utilizing proprioceptive inputs occurred was not directly assessed. As such, the interpretation of the results remains speculative, and further research is needed to validate these findings. Moreover, during the weight-shifting exercises in the A1, A2, and B phases, it is possible that larger weight shifts occurred in the B phase, where auditory feedback was used. However, this reflects clinical practice, where auditory feedback effectively guides patients to achieve consistent and sufficient weight shifting, resulting in greater improvements in postural control. Thus, we believe this supports the clinical usefulness of auditory feedback.

Although neither the examiner conducting the stabilometer tests nor the physical therapists performing the interventions were blinded, the interventions in each phase were generally consistent, apart from the use of auditory feedback. It cannot be ruled out that natural recovery over time contributed to the observed improvements in postural control. Additionally, since stabilometer measurements were conducted during every session, the potential influence of familiarity with the testing procedure cannot be excluded.

Nevertheless, significant improvements in postural control were observed in the B phase compared to the A1 phase. This suggests that weight-shifting exercises incorporating auditory feedback may enhance postural control in patients with BL. In the future, studies with larger sample sizes will be needed to verify these findings and to investigate which types of auditory feedback, such as continuous versus intermittent sounds, frequency adjustments, or the weight-shifting threshold at which sound is triggered, are most effective in improving postural control.

## Conclusions

This study suggests that weight-shifting exercises incorporating auditory feedback have the potential to contribute to improvements in postural control in patients with BL. The use of auditory feedback led to significant improvements in postural stability, even under eyes-closed conditions. To further validate these findings and elucidate the underlying mechanisms, future studies with larger sample sizes and objective assessments of proprioceptive reweighting and motor learning will be necessary.
